# Cultural adaptation and validation of the Computer Vision Syndrome Questionnaire in Colombia

**DOI:** 10.15649/cuidarte.3963

**Published:** 2025-08-19

**Authors:** Diana Carolina Silva Sánchez, Diego Fernando Rojas-Gualdrón, Natalia Eugenia Gómez Rúa, Elena Ronda Pérez, María del Mar Seguí

**Affiliations:** 1 Universidad CES, Medellín, Colombia. E-mail: silva.diana@uces.edu.co Universidad CES Medellín Colombia silva.diana@uces.edu.co; 2 Universidad CES, Medellín, Colombia. E-mail: dfrojas@ces.edu.co Universidad CES Medellín Colombia dfrojas@ces.edu.co; 3 Universidad CES, Medellín, Colombia. E-mail: ngomez@ces.edu.co Universidad CES Medellín Colombia ngomez@ces.edu.co; 4 Universidad de Alicante, Alicante, España. E-mail: elena.ronda@ua.es Universidad de Alicante Alicante España elena.ronda@ua.es; 5 Universidad de Alicante, Alicante, España. E-mail: mm.segui@ua.es Universidad de Alicante Alicante España mm.segui@ua.es

**Keywords:** Asthenopia, Occupational Health, Public Health, Validation Study, Psychometrics, Astenopía, Salud Ocupacional, Salud Pública, Estudio de Validación, Psicometría, Astenopia, Saúde Ocupacional, Saúde Pública, Estudo de Validação, Psicometria

## Abstract

**Introduction:**

Emerging risks at work may be caused by new information and communication technologies, such as computers. While they enhance productivity, computers can also cause new health issues in workers, such as Computer Vision Syndrome (CVS).

**Objective:**

To culturally adapt and evaluate the validity evidence of the Computer Vision Syndrome Questionnaire (CVS-Q©) for adult Colombian computer-using workers from the Rasch model approach.

**Materials and Methods:**

A quantitative, observational, cross-sectional, analytical study was conducted with a sample of 300 computer-using workers from a public university in Colombia in 2022. Rasch analyses were performed to fit response categories, items, and individuals; examine differential item functioning (DIF), dimensionality, and local independence of items; determine reliability; and produce the item-person map.

**Results:**

The CVS-Q (Colombia)© response format was adjusted to meet Linacre's guidelines for optimizing rating scale categories. All items showed infit and outfit mean-square values within the expected range. The scale was unidimensional, and the Rasch measurement explained 35.90% of the variance. Person reliability was 0.77, internal consistency was 0.88, and temporal stability reliability was 0.86.

**Discussion:**

The CVS-Q (Colombia)© assesses the frequency, intensity, and severity of sixteen signs and symptoms of CVS.

**Conclusion:**

The CVS-Q (Colombia)© is a valid and reliable scale for measuring the severity of CVS symptoms in Colombian workers.

## Resumen

**Introducción:**

Los riesgos emergentes en el trabajo pueden ser causados por las nuevas tecnologías de la información y la comunicación, como la computadora. Si bien esta mejora la productividad, también puede ocasionar nuevas alteraciones en la salud de los trabajadores como el Síndrome Visual Informático (SVI).

**Objetivo:**

Adaptar culturalmente y evaluar la evidencia de validez del Computer Vision Syndrome Questionnaire (CVS-Q©) en trabajadores colombianos adultos que utilizan computadoras desde la perspectiva del modelo de Rasch.

**Materiales y métodos:**

Estudio cuantitativo, observacional, transversal con intención analítica en una muestra de 300 trabajadores usuarios de computadoras de una universidad pública de Colombia en el año 2022. Se realizaron análisis de Rasch para ajustar las categorías de respuesta, ítems e individuos, examinar el funcionamiento diferencial de los ítems, la dimensionalidad e independencia local de los ítems, determinar la confiabilidad, y generar el mapa ítem-persona.

**Resultados:**

El formato de respuesta CVS-Q (Colombia)© se ajustó a los lineamientos de Linacre para la optimización de las categorías de la escala de valoración. Todos los ítems mostraron medias cuadráticas de infit y outfit dentro del rango esperado. La escala es unidimensional y la medida de Rasch explicó el 35,90% de la varianza. La confiabilidad de los individuos fue de 0,77, la consistencia interna fue de 0,88 y la confiabilidad de estabilidad temporal fue de 0,86.

**Discusión:**

El CVS-Q (Colombia)© evalúa la frecuencia, intensidad y severidad de dieciséis signos y síntomas del SVI.

**Conclusiones:**

El CVS-Q (Colombia)© es una escala válida y confiable para medir la gravedad de los síntomas del SVI en trabajadores colombianos.

## Resumo

**Introdução:**

Riscos emergentes no trabalho podem ser causados por novas tecnologias de informação e comunicação, como o uso do computador, que gera benefícios na produtividade, mas também novas alterações na saúde dos trabalhadores, como a Síndrome Visual Computacional.

**Objetivo:**

Adaptar culturalmente e avaliar as evidências de validade do Computer Visual Syndrome Questionnaire (CVS-Q©) em trabalhadores colombianos adultos que usam computadores a partir da perspectiva do modelo Rasch.

**Materiais e métodos:**

Estudo quantitativo, observacional, transversal com intenção analítica em uma amostra de 300 trabalhadores usuários de computador de uma universidade pública na Colômbia em 2022. Foram realizadas análises Rasch ajustando categorias de resposta, itens e indivíduos, funcionamento diferencial dos itens, dimensionalidade e independência local dos itens, confiabilidade e um mapa item-pessoa.

**Resultados:**

O formato de resposta do CVS-Q (Colômbia)© foi ajustado aos requisitos de otimização da função de Linacre, todos os itens apresentaram quadrados médios de Infit e Outfit na faixa esperada, a escala é unidimensional e a medida Rasch obtida do instrumento explicou 35,90% da variância. A confiabilidade para indivíduos foi de 0,77 com uma consistência interna de 0,88. A confiabilidade da estabilidade temporal foi de 0,86.

**Discussão:**

O CVS-Q (Colômbia)© é um questionário que avalia a frequência, intensidade e gravidade de dezesseis sinais e sintomas da Síndrome da Visão do Computador.

**Conclusões:**

O CVS-Q (Colômbia)© é uma escala válida e confiável para medir a gravidade dos sintomas da Síndrome da Visão do Computador em trabalhadores colombianos.

## Introduction

Computer Vision Syndrome (CVS) is also known as asthenopia, visual fatigue, digital eye strain, or digital visual fatigue. It refers to a set of visual and ocular signs and symptoms, including burning eyes, blurred or double vision, and tearing, among others, that result from focusing the eyes on a screen of any digital device for prolonged periods without rest^1^. 

CVS is a growing public health issue that can affect the quality of life and productivity of workers^2^–^8^. Almost sixty million people worldwide suffer from this condition, with one million new cases reported each year^9^. Symptoms affect almost 70% of all computer users^2^ and some studies conducted in Colombia using non-validated instruments and optometric assessments have indicated a high prevalence of CVS in workplace settings^10^–^13^. 

In Colombia, CVS has become a public health issue due to the increasing use of electronic devices across work and educational settings. A study conducted in Tunja found that the prevalence of CVS among university medical students was 84.4%, with a higher frequency in women (90%) than in men (75.80%)^14^. Additionally, a cross-sectional study at a public university in Cúcuta examined the association between CVS severity, healthy lifestyle habits, and the presence of dry eye syndrome in 300 employees who regularly use computers. The findings revealed significant correlations between CVS severity and factors such as physical activity, stress management, and dry eye symptoms^15^. 

This issue not only affects the quality of life of those experiencing it but also has implications for productivity and academic or professional performance. In other words, it becomes a broader social issue, particularly in contexts where screen use is unavoidable, but its effects could potentially be prevented or mitigated. 

Although a limited number of questionnaires have been developed to assess CVS^5^,^16^–^18^, the Computer Vision Syndrome Questionnaire (CVS-Q©) has been used in different studies. Originally developed in Spain, the CVS-Q© has demonstrated sensitivity and specificity values greater than 70%, good test-retest repeatability, and acceptable psychometric properties, as determined by Rasch analysis^19^. It has also been translated, adapted, and validated into other languages with favorable results. 

To obtain valid results regarding the magnitude of the severity of CVS symptoms in Colombia that contribute to collective decision-making for the execution of workplace prevention actions, a simple and valid instrument is required to complement occupational health surveys. Therefore, the objective of this study was to culturally adapt and validate the CVS-Q© for use among adult Colombian computer-using workers, employing the Rasch model approach. 

## Materials and Methods

This study was reported in accordance with the STROBE criteria^20^: 

**Study design and setting**

Quantitative, observational, cross-sectional study with analytical intent^21^, conducted at a public university in the city of San José de Cúcuta (Colombia) between November 2021 and December 2022. 

**Participants**

Computer-using employees at a Colombian university were invited to participate via email sent by the Human Resources Office. The inclusion criteria were: workers older than 18 years, born in Colombia, native Spanish speakers residing in Colombia, and having a minimum of one year of exposure to computers. Exclusion criteria included workers with a history of refractive surgery within the past year to correct myopia, hyperopia, astigmatism, or presbyopia; a history of cataract surgery; and workers receiving ophthalmological treatment (e.g., medications, ointments, eye drops, artificial tears) at the time of the study. 

**Variables and instruments**

The main variable measured was CVS severity measured through the CVS-Q©, a questionnaire designed and validated in Spain by Seguí et al.^19^ which assesses the frequency, intensity, and severity of sixteen CVS-related symptoms including burning, itching, foreign body sensation, tearing, excessive blinking, eye redness, eye pain, heavy eyelids, dryness, blurred vision, double vision, difficulty focusing close-up, increased sensitivity to light, colored halos around objects, feeling of worsening eyesight, and headache. 

The CVS-Q© includes three response options for frequency (0: never, 1: occasionally, 2: often/always) and two for intensity (1: moderate, 2: intense). According to the indications of the original questionnaire, severity is calculated by multiplying frequency by intensity, and then recoding the results as 0=0; 1 or 2=1; 4=2. It is worth noting that when analyzing data, an intensity score of "0: none" was assigned when the frequency was reported as "never," as this category is not included in the original questionnaire. Additionally, the Ocular Disease Surface Index (OSDI), developed by the Outcomes Research Group at Allergan Inc. (Irvine, California)^22^, was used. This 12-item questionnaire provides a rapid assessment of ocular irritation symptoms consistent with dry eye disease and their impact on vision-related functioning; it is a valid and reliable instrument for use in the Colombian population^23^. For data collection purposes, both instruments were implemented in a web-based application developed using Joomla and Bootstrap 4.50, with programming in HTML5, Java, and JavaScript, and a MySQL database. 

**Biases**

To ensure data quality, invitation emails were sent by the Human Resources Office to participants' institutional email accounts. Daily, the data storage platform was checked to verify the correct completion of the instruments. In addition, to minimize procedural bias, the invitation letter and informed consent form included explanations about the relevance of participating in the study, its contributions to public health, ethical considerations, and confidentiality of information. 

**Sample size**

A census was conducted via email, inviting all computer-using university employees to participate in the study and complete the instruments in a web application. To ensure high accuracy (99% confidence level), Linacre analysis was used as a reference, with a minimum sample size of between 250 participants and up to 20 respondents per item for instrument validation^24^. 

**Procedure for the cultural adaptation of the questionnaire**

**Figure 1:**
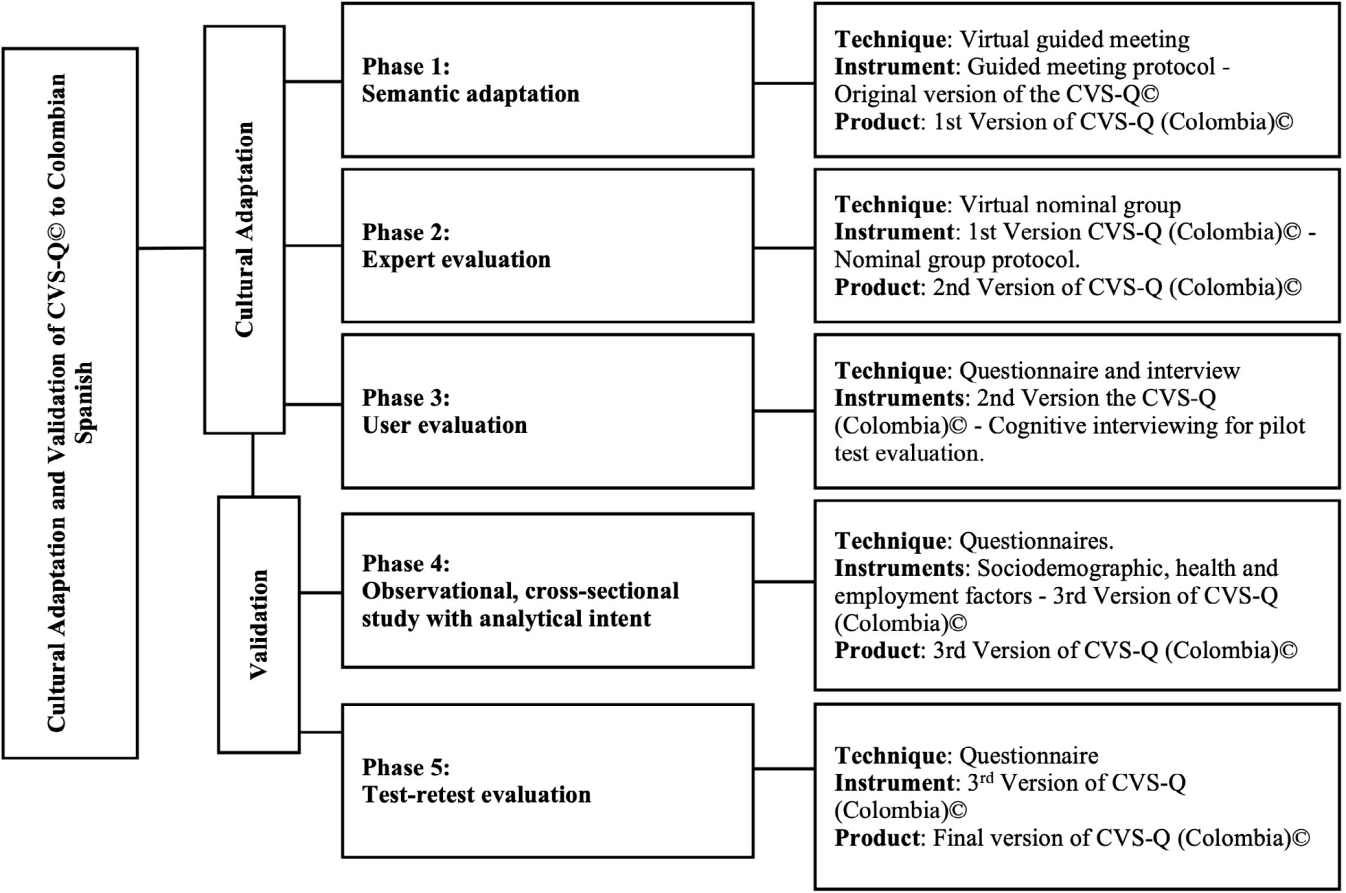
Process of adaptation and validation of the CVS-Q© to Colombian Spanish

Following the recommendations of the scientific literature and the developers of the CVS-Q© instrument^25^–^27^, the study methodology comprised the following phases (Figure 1). 

*CVS-Q: Computer Vision Syndrome Questionnaire *

In the first phase of semantic adaptation or cultural-linguistic adaptation^28^, a guided meeting was conducted via Zoom to discuss the terminology and expressions used in the original questionnaire. Five workers, users of digital devices, over the age of 18, from different work sectors, native to Colombia, and with a history of residence in Spain for more than two years, were invited. After grouping common responses, the first version of the CVS-Q (Colombia)© was developed. 

In the second phase, expert evaluation^29^,^30^, Zoom conferences were held to ensure the semantic, idiomatic, experiential, and conceptual equivalence of the first version. A specialist in Patient-Reported Outcome (PRO) questionnaires development, a specialist in preventive medicine, a public health expert, a professional in visual health and occupational health, two authors of the original questionnaire, and two additional public health experts, all with at least one year of experience, were summoned. After reaching a consensus among all the experts, the second version of the CVS-Q (Colombia)© was produced. 

In the third phase, the second version was evaluated by 50 Colombian-born workers who used digital devices. Participants were recruited through voluntary participation and snowball sampling. The objective was to identify errors, confirm the quality of the adaptation, verify practical aspects of its application, and identify the difficulties encountered during completion. This was achieved through a structured interview with open- and closed-ended questions conducted via Zoom. When at least 15% of the participants in this phase agreed to report issues with the questionnaire, adjustments were made^27^,^31^. 

**Questionnaire validation procedure**

Once the third version of the CVS-Q (Colombia)© was developed, validation was conducted through a cross-sectional epidemiological study of computer-using workers at a Colombian university, following the methodology used in the original questionnaire, which included Rasch analysis^32^–^34^. Finally, to assess temporal stability (test-retest) reliability, a subsample of 50 participants from the fourth phase completed the third version of the CVS-Q (Colombia)© between seven and fifteen days after the first administration. 

**Data analysis**

Categorical variables were described using absolute frequencies and percentages, while continuous variables were described using means and standard deviations. Considering the ordinal nature of the response options of the third version of the CVS-Q (Colombia)© and the authors' recommendations, the Rasch-Andrich rating scale guidelines were applied^35^. To obtain validity evidence, the following analyses were performed^36^. 

Fit of response categories. Compliance of the following Linacre's eight guidelines for optimizing rating scale categories^37^ was assessed: at least ten observations of each category, regular observation distribution, average measures advance monotonically with category, outfit mean-squares less than 2.00, Andrich thresholds advance, coherence measurement (categories imply measures [C->M] and measures imply categories [M->C]), Andrich thresholds advance ≥ 1.40 logits, and Andrich thresholds advance <5.00 logits. 

Fit of items and persons. It was assessed using infit and outfit mean squares with expected values between 0.50 and 2.00, and item-measure correlations with expected values >0.30. The assumption of local independence between items was verified by analyzing correlations between residuals, with expected values less than 0.70^35^. 

Differential item functioning (DIF). DIF was analyzed by age and sex, estimating the DIF contrasts and Mantel-Haenszel test probabilities. DIF was considered relevant when the contrast was ≥ 0.64 and p-value <0.05^26^. The interaction between the group and the attribute level was tested to determine whether the DIF was differential in nature (p-value <0.05) or non-differential (p-value ≥0.05). 

Dimensionality. Unidimensionality was assessed using principal component analysis (PCA) of the residual contrasts from the Rasch measurement, considering the following criteria: variance explained by the measure >40%, eigenvalue of the first residual component <2, and disattenuated correlation between the clusters of the first component residual >0.7. 

Reliability and Wright map. Reliability was estimated for items and persons, and was expressed through the separation index, which indicates the possible number of strata into which items and persons can be separated^38^. The Wright map is presented, and temporal stability reliability was assessed using test-retest analysis with Spearman's correlation coefficient, with a minimum acceptable value of 0.70^27^. 

Finally, dry eye symptoms were explored to analyze the similarity between responses regarding CVS severity using Spearman's correlation coefficient. Data were analyzed in Winsteps version 4.5.0^39^ and Stata version 16.1.^40^. 

**Ethical considerations**

The study was approved by the Ethics Committee of CES University (Project 960, Session 177, 2021) and conducted in accordance with the principles outlined in the Declaration of Helsinki^41^. For the first three phases, informed consent was obtained verbally; for the last two phases, consent was obtained electronically through the online application hosting the instrument. Likewise, the study complied with Law 1581 of 2012 on the protection of personal data. 

## Results

**Cultural adaptation of the questionnaire**

During the virtual meeting on semantic adaptation, the terminology and expressions used in the original CVS-Q© were discussed, and the first version of CVS-Q (Colombia)© was created. Adjustments were made to the introduction and items, replacing "itching" with "itching or stinging," "dryness" with "excessive dryness," and "colored halos around objects" with "colored reflections around objects." 

Subsequently, during the expert panel meeting, new adjustments were made to the introduction and items: "burning" was changed to "burning eyes", "itching or stinging" to "eye itching or stinging," "foreign body sensation" to "foreign body sensation in the eyes," "dryness" to "dry eyes" and "colored reflections around objects" for "colored reflections, lights or rings around objects." Although only six of the eight invited experts attended the meeting, the researchers and authors of the instrument considered the feedback sufficient to proceed with the process. 

In the third phase, 50 computer users evaluated the second version of the CVS-Q (Colombia)©. The completion time ranged from a minimum of 1'16'' to a maximum time of 7'16''. The instrument demonstrated good acceptability and comprehension, with 100% of participants reporting no difficulty understanding the instructions and not omitting any signs or symptoms from the questionnaire. Additionally, 10% (n=5) suggested including "fatigue" or "eye fatigue," 10% (n=5) proposed changes to the intensity levels, and 2% (n=1) reported difficulty filling in the frequency/intensity they experience any symptom. 

For the design of the third version of the CVS-Q (Colombia)©, feedback from 18% (n=9) of the participants was taken into account, as they proposed improvements to the virtual format of the instrument to enhance understanding. As a result, adjustments were made to the colors in the frequency and intensity columns, allowing the validation process to proceed. The final version of the CVS-Q (Colombia)© is available in Mendeley Data for download and consultation^42^. 

**Validation of the questionnaire**

***Participants***

Of the 434 workers who agreed to participate, 15.66% (n=134) were excluded for different reasons. Receiving ocular treatment (medications, ointments, eye drops, or artificial tears) at the time of the study was the most frequent reason for exclusion, at 50.74% (n=68). In addition, 14.05% (n=61) of workers had incomplete responses to the requested information in the instruments and were therefore excluded from the study. Other reasons for exclusion included a history of refractive surgery for correcting myopia, hyperopia, astigmatism, or presbyopia, combined with ongoing ocular treatment, and a history of cataract surgery with ongoing ocular treatment (3.73%, n=5). 

***Descriptive data***

The total study sample consisted of 300 participants. Ages ranged from 18 to 77 years, with a mean of 43.29 ± 12.89 years, and 57% were over 40 years old. Women accounted for 61.70% of the sample, and 57% were teachers (Table 1). The average daily use of a computer or other digital devices during the working day was 7.62 ± 4.23 hours. 

**Table 1:** Sociodemographic characteristics of the study participants n=300

Variable	% (n)
Sex	
Female	61.70 (185)
Male	38.30 (115)
Age (years)	
<40	43.00 (129)
≥40	57.00 (171)
Position	
Administrative	30.70 (92)
Professor	57.0 0(171)
Professor and administrative	10.70 (32)
Other	1.70 (5)
Time spent using the computer(hours/day)	
≤ 4	20.33 (61)
5-8	53.00 (159)
>8	26.66 (80)

Mantel-Haenszel test

**Main results**

***Fit of response categories***

The response structure of the categories corresponding to frequency, intensity, and severity was adjusted to Linacre's guidelines for optimizing rating scale categories. At least ten observations were obtained for each response category, ensuring a regular distribution of observations. In addition, the average measures advanced across all categories. The outfit mean-squares were within the expected range of 0.50-2.00; the Andrich thresholds showed advances across categories, and the coherence measurements (M->C, C->M) were greater than 40%. In conclusion, the fit of response categories shows sufficient alignment with the requirements of the Rasch model. (Table 2). 

***Fit of items and persons***

All the items met the infit mean-square criteria, with values ranging from 0.63 for "burning eyes" to 1.39 for "headache." Similarly, good outfit mean-squares were observed, ranging from 0.61 for "burning eyes" to 1.42 for "headache." The item-severity correlations ranged from 0.37 for "double vision" to 0.69 for "burning eyes" (Table 3). 

**Table 2:** Functioning of response categories for the CVS-Q (Colombia)© items

Category	Value	%(n)	Average measurement	Outfit MNSQ*	Andrich threshold		Coherence (%)	
					Extent	SE**	M-> C***	C-<M ****
Frequency								
Never	0	61.00 (2917)	-2.52	0.98			79	78
Occasionally	1	34.00 (1625)	-1.00	0.86	-1.40	0.04	61	69
Sometimes/Always	2	5.00 (258)	-0.05	1.27	1.40	0.07	61	11
Intensity								
None	0	61.00 (2915)	-2.96	1.00			78	80
Moderate	1	36.00 (1718)	-1.29	0.92	-1.82	0.04	66	68
Intense	2	3.00 (167)	0.15	1.11	1.82	0.09	55	10
Severity								
Never	0	61.00 (2917)	-3.15	0.99			78	81
Occasionally (1) – Moderate (1) Occasionally (1) – Intense (2) Sometimes/Always (2) – Moderate (1)	1	37.00 (1755)	-1.42	0.93	-2.00	0.04	68	68
Sometimes/Always (2) – Intense (2)	2	3.00 (128)	0.01	1.14	2.00	0.10	40	5

*MNSQ: Mean-square; **SE: standard error; ***M->C: the measure implies the category; ****C->M: the category implies the measure. Mantel-Haenszel test.

**Table 3:** Item location and fit statistics of the CVS-Q (Colombia)©

Item severity	Location	SE	MNSQ		Total item Correlation
			Infit	Outfit	
1. Burning eyes	-0.71	0.13	0.63	0.61	0.69
2. Eye itching or stinging	-0.99	0.13	0.95	0.96	0.58
3. Sensation of a foreign body in the eyes	0.22	0.14	0.99	0.96	0.56
4. Tearing	-0.42	0.13	1.00	1.07	0.57
5. Excessive blinking	0.84	0.15	0.87	0.79	0.55
6. Eye redness	-0.03	0.14	0.97	0.97	0.57
7. Eye pain	0.65	0.15	0.86	0.70	0.60
8. Heavy eyelids	0.26	0.14	0.93	0.88	0.57
9. Dry eyes	-0.19	0.13	1.05	1.06	0.59
10. Blurred vision	-0.71	0.13	0.81	0.77	0.68
11. Double vision	1.93	0.19	1.22	1.21	0.37
12. Difficulty focusing close-up	-0.35	0.13	1.30	1.26	0.54
13. Increased sensitivity to light	-0.45	0.13	0.91	0.87	0.65
14. Reflections, lights, or colored rings around objects	0.6	0.15	1.09	1.17	0.50
15. Feeling of worsening eyesight	0.5	0.14	1.00	0.84	0.62
16. Headache	-1.15	0.13	1.39	1.42	0.52

MNSQ: Mean-square; SE: Standard error. Mantel-Haenszel test.

 According to the person-fit statistics, an infit underfit was observed in 3.3% of participants (mean=0.99, SD=0.38), and an outfit underfit was observed in 4.70% (mean=0.98, SD=0.48).

***Differential item functioning (DIF)***

 According to the DIF contrasts between the measurements of the CVS-Q (Colombia)© items by age and sex, type C DIF was identified for the items "dry eyes" and "difficulty focusing close-up" according to age (reference: ≥40 years), and for "tearing," "eye redness," and "headache" according to sex (reference: female). Type D DIF was observed for "blurred vision" and "headache" according to age, and for "eye pain," "increased sensitivity to light," and "reflections, lights or colored rings around objects” according to sex.

 Statistically significant DIF by age was found for the items "dry eyes," "blurred vision," "difficulty focusing close-up," and "headache." By sex, statistically significant DIF was observed for "tearing," "eye redness," "increased sensitivity to light," and "headache." (Table 4). 

**Table 4:** Differential item functioning of the CVS-Q (Colombia)© items among computer-using workers

Item severity	Age						Sex					
	<40		≥40		Cont***	p-value	Male		Female		Cont***	p-value
	β*	SE**	β*	SE**			β*	SE**	β*	SE**		
1. Burning eyes	-0.83	0.20	-0.62	0.17	-0.21	0.43	-0.93	0.21	-0.57	0.17	-0.36	0.18
2. Eye itching or stinging	-1.06	0.20	-0.93	0.17	-0.13	0.62	-1.10	0.21	-0.92	0.16	-0.18	0.50
3. Foreign body sensation in the eyes	0.06	0.21	0.34	0.18	-0.27	0.33	0.04	0.22	0.33	0.18	-0.29	0.31
4. Tearing	-0.50	0.20	-0.36	0.17	-0.15	0.58	-0.84	0.21	-0.15	0.17	-0.69	0.01
5. Excessive blinking	0.65	0.23	1	0.2	-0.35	0.25	0.76	0.25	0.90	0.19	-0.14	0.65
6. Eye redness	-0.21	0.21	0.11	0.18	-0.31	0.26	-0.58	0.21	0.33	0.18	-0.91	0.00
7. Eye pain	0.54	0.23	0.72	0.19	-0.18	0.54	0.95	0.26	0.49	0.18	0.46	0.15
8. Heavy eyelids	0.20	0.22	0.30	0.18	-0.1	0.72	0.52	0.24	0.12	0.17	0.4	0.17
9. Dry eyes	0.20	0.22	-0.44	0,17	0.64	0.02	-0.01	0.22	-0.29	0.17	0.28	0.32
10. Blurred vision	-0.34	0.21	-0.96	0.17	0.63	0.02	-0.80	0.21	-0.65	0.16	-0.15	0.58
11. Double vision	1.71	0.28	2.11	0.26	-0.40	0.29	1.71	0.30	2.07	0.24	-0.36	0.35
12. Difficulty focusing close-up	0.49	0.22	-0.88	0.17	1.37	<0.01	-0.49	0.21	-0.26	0.17	-0.22	0.41
13. Increased sensitivity to light	-0.50	0.20	-0.41	0.17	-0.09	0.74	-0.06	0.22	-0.68	0.16	0.62	0.03
14. Reflections, lights or colored rings around objects	0.39	0.22	0.76	0.19	-0.37	0.21	0.88	0.25	0.46	0.18	0.43	0.17
15. Feeling of worsening eyesight	0.65	0.23	0.40	0.18	0.24	0.41	0.58	0.24	0.46	0.18	0.12	0.69
16. Headache	-1.48	0.19	-0.9	0.17	-0.58	0.03	-0.53	0.21	-1.52	0.16	0.99	0.00

*β: extent; **SE: standard error; ***Cont: contrast. Mantel-Haenszel test.

***Dimensionality***

The Rasch measurement analysis of the CVS-Q (Colombia)© explained 35.9% of the total variance in the scores of the 16 items. The first contrast accounted for 8.20% of the residual variance, with an eigenvalue of 2. The disattenuated correlation between the clusters of the first contrast was 0.73. Therefore, when analyzing the first contrast, a group of visual signs and symptoms and another of ocular symptoms were identified: 1) feeling of worsening eyesight, blurred vision, difficulty focusing close-up, reflections, lights or colored rings around objects, and double vision vs. 2) eye pain, eye burning, eye redness, headache, eye itching or stinging, heavy eyelids, excessive blinking, foreign body sensation in the eyes, dry eyes, increased sensitivity to light, and tearing. 

***Reliability and Wright map***

**Figure 2:**
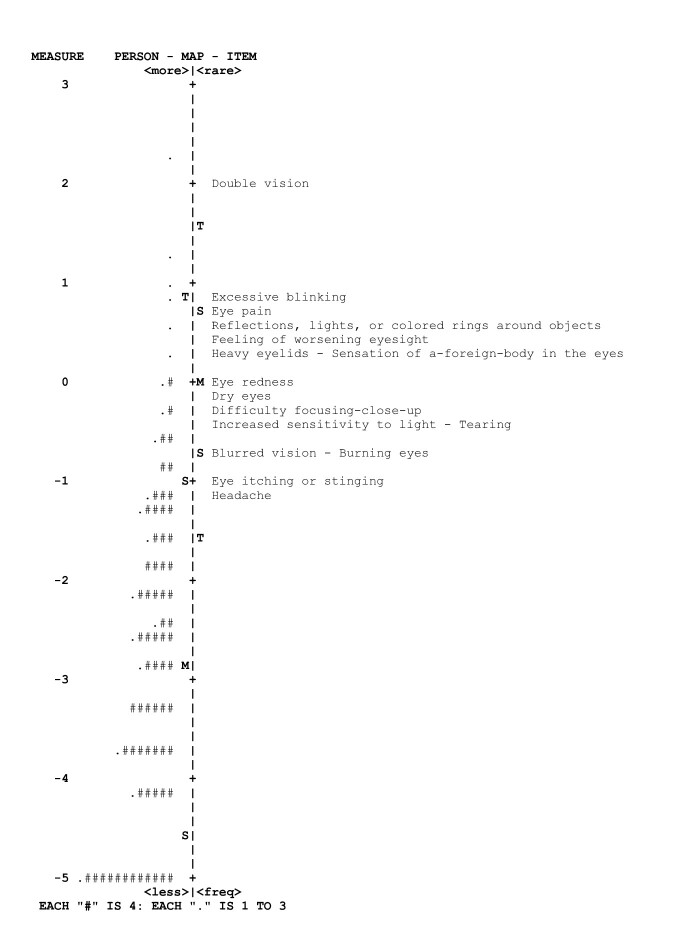
Wright map

Person reliability was 0.77, with an internal consistency of 0.88. The floor effect was 11.70%. In the Wright map, items that have values between 2 and 4 logits corresponded to symptoms that tend to provide clearer evidence of high CVS severity: foreign body sensation; heavy eyelids; feeling of worsening eyesight; reflections, lights or colored rings around objects; eye pain; excessive blinking, and double vision (4 logits) (Figure 2). The test-retest reliability or temporal stability, assessed over an average interval of 9 days, was 0.86.

Finally, analysis of the similarity in the responses with the Ocular Disease Surface Index (OSDI) yielded a moderate correlation of 0.64.

## Discussion

The main objective of this study was to culturally adapt and evaluate the validity evidence of the CVS-Q©, originally developed in Spanish (Spain), for use in Spanish (Colombia), resulting in the CVS-Q (Colombia)©. To date, the original CVS-Q© has been translated and culturally adapted into English, Slovak, Italian, and Persian^34^,^43^–^44^, following the methodology proposed by Beaton et al.^26^. However, since a translation process was not required for the present study, the guidelines provided by the authors of the original questionnaire along with some recommendations from Beaton et al. were followed. Regarding cultural adaptation, during the first two phases, changes were agreed upon in the introduction and some items, considering the linguistic and contextual differences between the two countries, which can influence the validity of a questionnaire when applied in occupational health settings^25^,^45^–^47^. The profiles of the experts invited to participate were similar to those reported by Seguí et al.^34^, Mikulášová et al.^43^, Seguí-Crespo et al.^48^, and Qolami et al.^44^. 

The third version of the CVS-Q (Colombia)© turned out to be identical to the version approved by the committee of experts, as it had good acceptability and comprehension among the 50 computer users, a situation similar to the adaptation process for the Italian version^48^. However, in the case of adaptation to Slovak, two changes were introduced in the questionnaire because more than 15% of the respondents recommended adding the phrase "in the eye" to the symptom "foreign body sensation," and the word "eyes" for four more symptoms^43^, recommendations given at the meeting of experts in Colombia. 

In relation to the average completion time of the questionnaire during the cultural adaptation process, the results obtained were reasonable and similar to those of the original version (mean: 2 minutes)^34^, and comparable to the Italian version (1.57± 1.17 minutes)^48^, Slovak version (2.48 ± 1.17 minutes)^43^, Colombian version (2.58 ± 1.18 minutes), and Persian version (3.10 ± 1.70 minutes)^44^ (mean ± standard deviation). For the validation process, the study followed Messick's proposal of facets of validity evidence (aspects of construct validity)^49^, as well as the recommendations of the Scientific Advisory Committee of the Medical Outcomes Trust^50^. 

Substantive aspect. The response categories to the items showed sufficient fit and consistency. This indicates that, when completing the questionnaire, workers rated the symptoms with the level of detail assumed by the instrument. This occurs when assessing the severity of certain CVS symptoms, such as double vision (diplopia), which, according to some authors, including Erdinest and Berkow^51^, is not a specific symptom of SVI. In contrast, Akinbinu and Mashalla^52^ argue that, in the absence of neurological lesions or diseases affecting the ocular muscles, diplopia can be attributed to extraocular muscle fatigue resulting from prolonged exposure to glare from the computer screen without breaks. 

Content aspect. The item fit and person fit, as well as the observed item-measure correlations, suggest that the operationalization of the CVS severity across the 16 items is appropriate for the latent attribute. This suggests that the response pattern according to the measurement level of the items and the severity of workers' CVS symptoms, is consistent with Rasch model assumptions. 

Structural aspect. The original CVS-Q© was designed to measure a single dimension, and the results for the CVS-Q (Colombia)© suggest that a unidimensional structure is appropriate. Regarding the residuals of the proposed measure, no relevant factors were identified in the explained variance. However, analysis of the first contrast revealed two distinct groups: visual symptoms and ocular symptoms, similar to the classification reported by Blehm et al.^53^, who categorized CVS symptoms into visual, ocular, asthenopic, light-related, and musculoskeletal symptoms. 

Generalizability aspect. Both person reliability and internal consistency were above 0.77. Although the generalization of the CVS-Q (Colombia)© by sociodemographic subgroups was affected by the presence of DIF in nine items, this had a significant impact on only a small proportion of workers. Overall, the evidence was not sufficient to warrant item removal. For comparisons of CVS severity between sociodemographic groups using this questionnaire, it is recommended to consider the possibility of excluding items with differential item functioning identified in this study. 

According to the validation results obtained through Rasch analysis for the original, Italian, and Colombian versions of the questionnaire, all demonstrated acceptable item and person fit to the model, as well as reliability. In the case of the original and Italian versions, sensitivity, specificity, and AUC values were also analyzed, with better results in the Italian version (sensitivity = 75% vs. 80%; specificity = 70.20% vs. 83.10%; AUC = 0.82 vs. 0.87), indicating a marginally better internal performance for this version^54^. 

The original CVS-Q© has also been validated in Persian for the Iranian working population^44^ and in Peruvian Spanish for healthcare professionals^55^ using factor analysis. According to the results, between these two versions (Persian vs. Peruvian Spanish), the Peruvian version obtained slightly better results in specificity and the intraclass correlation coefficient (ICC) between severity scores from the test and retest: Sensitivity = 81.1% vs. 72.22%; specificity = 69.20% vs. 100%; ICC = 0.81 vs. 0.85. 

Regarding internal consistency, Cronbach's alpha in this study was acceptable (0.88) and comparable to values reported in the literature, which range from 0.76 to 0.93: the original version (0.78)^34^, the Italian (0.76)^54^, the Persian (0.80)^44^, and the Peruvian (0.93)^55^. As in the Colombian study, none of these validations required item removal due to execution difficulties in any of these studies. 

Finally, the Colombian test-retest analysis of the CVS-Q revealed good temporal stability (0.86), comparable to results reported for the original version (ICC = 0.80; 95% CI: 0.67-0.88)^34^, Italian (ICC = 0.72; 95% CI: 0.49-0.85)^54^, Peruvian (ICC = 0.85; 95% CI: 0.77-0.90)^55^, and Persian (ICC = 0.81; 95% CI)^44^ versions. 

## Conclusions

The CVS-Q (Colombia)© was culturally adapted and validated for Colombian Spanish. This study provides a valid and reliable instrument for assessing the severity of CVS among computer-using employees at a university in Colombia, thereby contributing to the collection of information to address a social problem affecting this population through the appropriate application of an instrument. 

From a public health perspective, the need to encourage behaviors that promote visual and ocular health is clear, as well as promoting preventive CVS interventions for computer-using workers through context-specific programs. It is convenient for workers to be informed about this syndrome before its onset or the worsening of its consequences, which can hinder daily work tasks. 
